# Triiodothyronine (T3) Induces Limited Transcriptional and DNA Methylation Reprogramming in Human Monocytes

**DOI:** 10.3390/biomedicines10030608

**Published:** 2022-03-04

**Authors:** Rebecca Shepherd, Bowon Kim, Richard Saffery, Boris Novakovic

**Affiliations:** 1 Molecular Immunity, Infection and Immunity Theme, Murdoch Children’s Research Institute, Parkville, VIC 3052, Australia; rebecca.shepherd14@mcri.edu.au (R.S.); bowon.kim@mcri.edu.au (B.K.); richard.saffery@mcri.edu.au (R.S.); 2 Department of Paediatrics, The University of Melbourne, Parkville, VIC 3052, Australia

**Keywords:** monocytes, thyroid hormone, T3, triiodothyronine, DNA methylation, transcriptome, epigenetics, EPIC

## Abstract

Thyroid hormones have immunomodulatory roles, but their effects on the transcriptome and epigenome of innate immune cell types remain unexplored. In this study, we investigate the effects of triiodothyronine (T3) on the transcriptome and methylome of human monocytes in vitro, both in resting and lipopolysaccharide (LPS)-stimulated conditions. In resting monocytes, 5 µM T3 affected the expression of a small number of monocyte-to-macrophage differentiation-associated genes, including *TLR4* (*p*-value < 0.05, expression fold change >1.5). T3 attenuated a small proportion of monocyte-to-macrophage differentiation-associated DNA methylation changes, while specifically inducing DNA methylation changes at several hundred differentially methylated CpG probes (DMPs) (*p*-value < 0.05, Δβ > 0.05). In LPS-stimulated monocytes, the presence of T3 attenuated the effect of 27% of LPS-induced DMPs (*p*-value < 0.05, Δβ > 0.05). Interestingly, co-stimulation with T3 + LPS induced a unique DNA methylation signature that was not observed in the LPS-only or T3-only exposure groups. Our results suggest that T3 induces limited transcriptional and DNA methylation remodeling in genes enriched in metabolism and immune processes and alters the normal in vitro LPS response. The overlap between differentially expressed genes and genes associated with DMPs was minimal; thus, other epigenetic mechanisms may underpin the expression changes. This research provides insight into the complex interplay between thyroid hormones, epigenetic remodeling, and transcriptional dynamics in monocytes.

## 1. Introduction

Cells of the innate immune system, primarily monocytes, can form nonspecific memory in response to a range of microbial compounds, danger signals, and metabolic precursors [1,2]. This memory, known as trained immunity, is underlined by metabolic, epigenetic, and transcriptional reprogramming [3,4,5]. Epigenetic mechanisms include DNA methylation, histone tail modifications, long non-coding RNA, and micro RNA, which can influence transcription in a context-specific manner [6]. DNA methylation is highly dynamic during cell differentiation [7], but is also susceptible to changes in response to environmental factors, including hormone exposure [8].

Accumulated evidence indicates a complex interplay between the immune system and the neuroendocrine system, with endogenous and exogenous hormone exposure altering innate immune function [9]. To date, there has been a strong focus on reproductive hormones and glucocorticoids, but in recent years, thyroid hormones have been proposed to have an immunomodulatory role [10,11,12,13,14,15,16]. This has been specifically highlighted in studies of murine macrophages in the context of response to lipopolysaccharide (LPS) [10,11].

The thyroid gland, under the regulation of thyroid-stimulating hormone (TSH), predominantly produces thyroxine (T4), accounting for ~80% of hormone output, and triiodothyronine (T3), accounting for ~20% [17]. T3 is considered more biologically active and most of the prohormone thyroxine (T4) is peripherally converted to T3 [17,18]. The thyroid hormone receptor (TR) recognizes both thyroid hormones, but has an affinity for T3 more than 20 times higher than for T4 [18]. Canonical thyroid hormone signaling involves direct interaction with the TR and the regulation of downstream genes containing thyroid response elements (TREs) [19]. Ligand-bound TRs can also induce rapid PI3K signal transduction in a non-canonical manner, accounting for, at least in part, the rapid effects of T3, independent of altered gene expression [20]. Additionally, thyroid hormones can bind the extracellular domain of integrin αVβ3, resulting in signaling through the MAPK and ERK1/2 pathways [21]. TR expression has been identified in human neutrophils [22], uterine NK cells, and macrophages [12,23], with various TR isoforms differentially expressed in a cell-specific manner. The expression of TR in human macrophages and monocytes remains controversial; however, studies have highlighted the effects of thyroid hormones on these cell types [24,25].

A range of study types has indicated that thyroid hormones have effects on monocyte and macrophage function and phenotype, including cytokine production, reactive nitrogen species production, phagocytosis, and the inflammatory response to LPS (reviewed in [26]). DNA methylation is one epigenetic mechanism by which gene expression and subsequent monocyte identity and function are regulated [27,28]. The specific effects of thyroid hormones on the monocyte methylome remains understudied, but the outcome of such studies may aid in understanding how thyroid hormones influence monocyte function. Indeed, if thyroid hormones induce DNA methylation changes, these changes may be exploited to stratify patients according to their thyroid state [29].

In the current study, we characterize the in vitro effects of T3 on genome-wide DNA methylation (DNAme) and transcriptomes in resting and LPS-stimulated human monocytes using an established model of monocyte plasticity [30]. The results of this study aid the understanding of the effects of T3 on monocyte methylation, gene expression, and inflammation, which is of interest in both a physiological context and in the context of thyroid dysfunction, such as hyperthyroidism and hypothyroidism.

## 2. Materials and Methods

### 2.1. Sample Source

Human adult buffy coats (*n* = 3) of approximately 60 mL in volume were obtained from the Australian Red Cross (ARC agreement deed: 18-10VlC-08) with ethical approval (Human Research Ethics Committee (HREC) reference number: HREC/43573/RCHM-2018-150854(v2)). As donors were anonymous and limited donor information was provided, donor sex was determined using expression of the *XIST* gene; all three donors were assigned as male.

### 2.2. Monocyte Isolation

Buffy coat samples (60 mL) were distributed across tubes, at 10 mL/tube, and then topped up to 35 mL with PBS. Peripheral blood mononuclear cells (PBMCs) were isolated from diluted buffy coats through a Ficoll-Paque gradient, with 15 mL Ficoll-Paque (Sigma, Victoria, Australia, GE17-1440-02) underlaid per tube, followed by centrifugation at 1700 rpm for 25 min (no brake, room temperature). PBMCs were collected from the cloudy interphase layer, washed once with PBS, pooled for each donor, and washed a second time with PBS. The PBMCs were then counted using an automated cell counter and resuspended in RPMI. Monocytes were isolated from PBMCs using a hyperosmotic Percoll gradient (prepared with 9.7 mL Percoll (Sigma, Victoria, Australia, P1644), 8.3 mL RO H_2_O, and 2.0 mL 1.5 M NaCl for every 200 × 10^6^ cells) with 3 mL PBMC-containing medium overlaid on 10 mL hyperosmotic Percoll and centrifuged at 2350 rpm for 30 min (no brake, room temperature). Monocytes were collected from the cloudy interphase layer, washed once with RPMI medium, and counted. The monocytes were resuspended in RPMI and 3 mL of monocyte-containing medium was overlaid on 10 mL of isosmotic Percoll (prepared with 8.3 mL of Percoll, 9.7 mL of RO H_2_O, 2.0 mL of 1.5 M NaCl, 10 mL for every 200 × 10^6^ cells). The monocyte pellet was then resuspended in RPMI and counted on an automated cell counter.

### 2.3. Monocyte Stimulation

Isolated monocytes were seeded in 6-well plates (9.6 cm^2^/well) at a density of 2 × 10^6^ monocytes/well in 2 mL RPMI culture medium for 1 h at 37 °C, with visual confirmation of attachment by light microscopy. The seeding medium was then aspirated from the plate and the attached monocytes were treated with either RPMI medium, 5 µM triiodo-L-thyronine (T3) (Sigma, Victoria, Australia, T0281), 10 ng/mL lipopolysaccharide (LPS) (*E. coli* O55:B5, Sigma, Victoria, Australia, L2880), or a costimulation of 5 µM T3 and 10 ng/mL LPS (T3 + LPS) (with monocytes pre-exposed to T3 for 1 h prior to the addition of LPS) for 4 h or 24 h at 37 °C. All treatments were administered at a volume of 2 mL per well, with RPMI used as the diluent. Separate wells were used for the subsequent RNA and DNA collection. Monocytes for subsequent RNA extraction were collected in 500 µL Trizol and stored at −80 °C. Monocytes for subsequent DNA extraction were collected in 500 µL of ATL buffer (QIAGEN, Victoria, Australia, 56304) and stored at −30 °C. In addition to 4 h and 24 h treatments, untreated monocytes were also collected for DNA and RNA extraction following seeding as a time zero (0 h) control.

### 2.4. RNA Extraction and Sequencing

Monocytes stored in Trizol were thawed on ice and 100 µL of chloroform was added and samples were mixed well by inversion. The samples were spun at 12,000 rcf for 15 min at 4 °C and the upper transparent phase was transferred into a new 2 mL tube. Then, 300 µL of 70% ethanol was added and mixed well, and samples were then aliquoted into the RNeasy mini spin column (QIAGEN, Victoria, Australia, 74106). RNA was then extracted using the RNeasy mini kit (QIAGEN, Victoria, Australia, 74106), as per protocol, and eluted in 30 µL of RNase-free water. RNA concentration was quantified using the NanoDrop 1000 spectrophotometer. Prior to sequencing, the RNA samples were run on the Agilent TapeStation to confirm suitable quality and quantity for sequencing.

RNA was prepared for sequencing using the TruSeq Stranded mRNA kit (Illumina, Victoria, Australia, RS-122-2101) as per the manufacturer’s protocol. Prepared mRNA samples were sequenced on the Illumina NovaSeq 6000 sequencing system with 100 bp paired ends. The RNAseq output files containing the sequence reads (.fastq files) were aligned to the reference transcriptome (Homo sapiens GRCh37.70) using Bowtie1. Aligned reads were quantified and normalized using the MMSEQ package [31]. Only protein-coding genes were considered in downstream analysis.

### 2.5. RNA Sequencing Analysis

Differentially expressed genes (DEGs) were identified using the DESEQ2 package [32] in RStudio with donor ID (*n* = 3) included in the model. DEGs were identified as those with a DESEQ2 log_2_ fold change (counts) of >0.58 or <−0.58 (equating to a fold change of 1.5), an unadjusted *p*-value of <0.05, and a mean reads per kilobase of transcript per million mapped reads (RPKM) value of >1. To minimize noise, genes for which the DESEQ2 log_2_ fold change showed an opposite direction to the log_2_ fold change in mean RPKM values were excluded. Table 1 outlines RNAseq comparisons and cut-offs used to identify DEGs in more detail. Considering that monocytes begin to differentiate in culture rapidly due to endogenous M-CSF production, and that monocytes exhibit distinct early (4 h) and late (24 h) responses to medium (early and late differentiation programming) and LPS (early and late LPS response) [4], we decided *a priori* to investigate pairwise comparisons of time points in models containing two time points instead of fitting a linear or higher-order relationship between time and gene expression (or methylation) across all three time points. However, it is worth noting that a time series model could have been applied to encompass all three time points.

### 2.6. DNA Extraction and DNA Methylation Array

Monocytes stored in ATL buffer were thawed and lysed with proteinase K for 2 h, and DNA was extracted using the QIAamp^®^ DNA Mini spin kit (QIAGEN, Victoria, Australia, 56304) and eluted in 50 µL of elution buffer. Gel electrophoresis was used to confirm successful DNA extraction. Genomic DNA extracted from monocytes was plated into 96-well plates at a concentration of 50 ng/µL with a volume of 15 µL per well, and sent to Erasmus Medical Centre, Netherlands, for sodium bisulfite conversion and genome-wide methylation analysis using the Illumina Infinium Methylation EPIC Array, which measures DNA methylation across 850,000 CpG sites (referred to as EPIC ‘probes’); these span promoter regions, gene bodies, and ENCODE-assigned distal regulatory elements. Briefly, bisulfite conversion involves the treatment of denatured DNA with sodium bisulfite, which deaminates unmethylated cytosines (converting them to uracils), whilst methylated cytosines remain unchanged. An output of this array used in downstream analysis is the beta (β) value, which is an estimation of methylation computed by the ratio of intensities between methylated and unmethylated alleles at the respective locus.

### 2.7. DNA Methylation Data Analysis

Raw IDAT files were processed using the MissMethyl and minfi packages for R [33,34]. All samples had a good quality score (mean detection *p*-value of <0.01). Data were normalized for both within- and between-array technical variation using SWAN (Subset-quantile Within Array Normalization) [35]. Probes with poor average quality scores (detection *p*-value > 0.01), those that overlap a SNP at their CG site, and cross-reactive probes were removed from downstream analysis [36]. Probes on X and Y chromosomes were also excluded. Donor samples were confirmed as matched using a set of SNPs in the minfi package, and no mismatches or mislabeled samples were identified. Differential methylation analysis by linear regression modeling was performed using the limma package [37]. Differentially methylated probes (DMPs) were those with an unadjusted *p*-value of <0.05 and a change in methylation (delta beta, Δβ) of >0.05 or <−0.05, equating to a change in methylation of >5%. Table 1 outlines RNAseq comparisons and cut-offs to identify DMPs in more detail.

Differentially methylated regions (DMRs), which are regions of DNA containing multiple CpGs showing correlative methylation changes, were identified using the DMRcate tool in R (*p*-value < 0.05, minimum three CpGs, with at least one CpG showing a Δβ of >0.05 or <−0.05) [38] and Bedtools were used to intersect DMRs with individual EPIC probes [39]. The comparisons of groups used for DMR identification were the same as those for DMP identification (Table 1).

### 2.8. Motif Analysis and Gene Ontology

DMPs were assigned to the nearest gene within 1 megabase (1 Mb) using the GREAT tool [40]. Transcription factor (TF) binding motif (TFBM) analysis was performed on gene lists, or genomic sequences (−100 bp to +100 bp) around DMPs using HOMER (http://homer.ucsd.edu/homer/, accessed on 20 January 2022). Motifs were considered enriched if they met the criteria of a *p*-value of <0.05, a fold change in abundance of >1.5 (% Target sequences containing the motif/% Background sequences containing the motif), a difference in abundance of >5% (% Target sequences containing the motif—% Background sequences containing the motif), and minimum of two targets in the list containing the motif. Motifs with no meaningful TF expression in the monocyte transcriptome (i.e., genes in which all treatment groups showed a log_2_ mean RPKM < 0) were excluded. Enriched biological process (BP) terms and KEGG pathways were identified using HOMER (*p*-value < 0.05, minimum two genes in term).

### 2.9. Data Statement and Availability

The data sets generated and analyzed for the current study were deposited in the Gene Expression Omnibus repository with the SuperSeries accession number GSE180694. The SubSeries accession numbers are GSE180693 for RNA sequencing and GSE180692 for DNA methylation data.

## 3. Results

### 3.1. T3 Alters a Subset of Monocyte-to-Macrophage Differentiation DNA Methylation Changes in Promoters of Metabolism Genes

**Figure 1:**
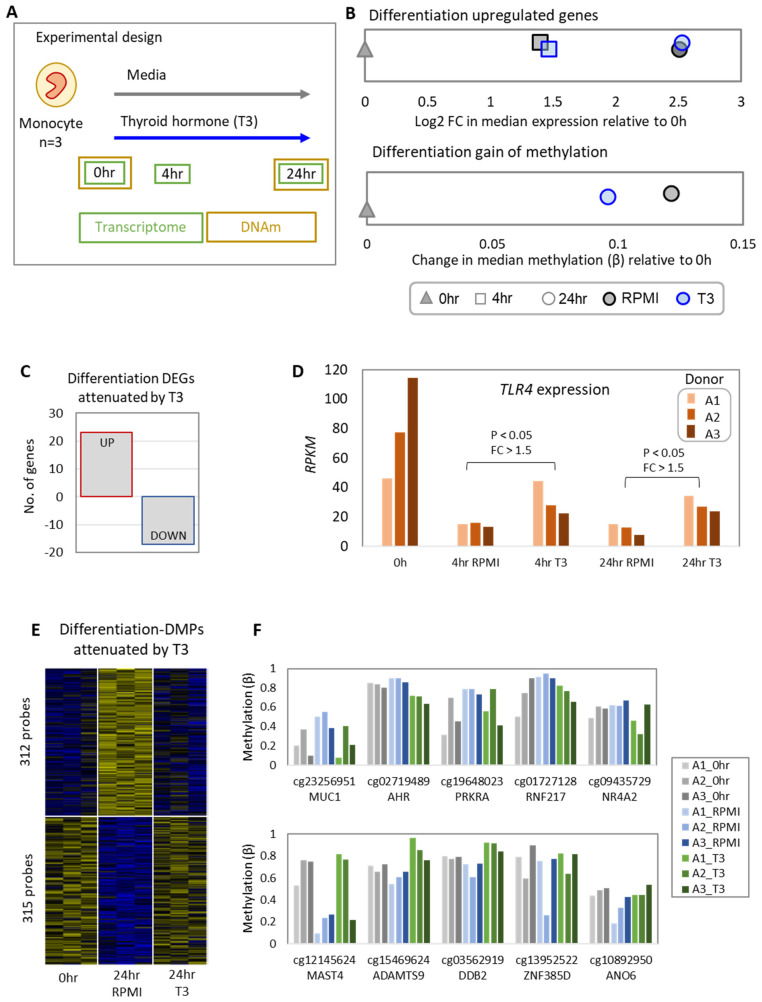
T3 impedes a subset of methylation changes associated with 24 h monocyte-to-macrophage differentiation. (**A**) Isolated human monocytes (*n* = 3) were exposed in vitro to 5 µM of triiodothyronine (T3) or RPMI medium for 4 h and 24 h for RNAseq and/or DNA methylation. (**B**) Top plot shows axis of strongly upregulated differentiation-associated genes (FC counts > 1.5, adj *p*-value < 0.05), with T3 having no effect on the overall trajectory. Figure was made by calculating the median expression RPKM for each treatment group mean, then calculating the fold change of this median relative to the 0 h median (median treatment/median 0 h). The log-transformed fold change for each treatment is plotted on a single axis (x axis). Median was calculated from RPKM values of all upregulated genes in the list. Here, we can see that at both time points, T3 has no effect on the overall differentiation trajectory. Bottom plot shows axis of most strongly differentiation-associated gain of methylation probes (24 h RPMI vs. 0 h Δβ > 0.10, *p*-value < 0.05), with T3 having an abrogating effect. Here, the x axis shows the change in median beta value (β) in the treatment (24 h T3 or 24 h RPMI) compared to the 0 h group. Median was calculated from all β values of DMPs in the list. (**C**) T3 had a limited effect on attenuating expression of differentiation-associated genes, with 23 differentiation-downregulated genes upregulated in the presence of T3, and 17 differentiation-upregulated genes downregulated in the presence of T3. (**D**) *TLR4* is one example of a differentiation gene upregulated in the presence of T3 relative to the time-matched control, with a fold change in mean expression of >1.5 and a *p*-value of <0.05. Bar plots are individual donor RPKM values. (**E**) T3 attenuates the effect of differentiation in 4.3% (312/7210) of differentiation–gain-of-methylation probes and 2.7% (315/11,927) of differentiation–loss-of-methylation probes (Z-scored individual donor β values for 0 h, 24 h RPMI, and 24 h T3 treatments) (**F**) Top gain-of-methylation (top plot) and loss-of-methylation (bottom plot) differentiation DMPs attenuated by T3. Bar plots are individual donor methylation (β) values in the 0 h, 24 h RPMI, or 24 h T3 groups.

Monocytes were cultured for 24 h in RPMI medium alone or with 5 µM T3, and molecular profiling was performed at attachment (0 h; RNA and DNAme), 4 h (RNA), and 24 h (RNA and DNAme) (Figure 1A). In the first 24 h of culture, monocytes start to differentiate to macrophages in the presence of endogenous M-CSF [30]. We assessed the differentiation-associated changes in DNA methylation changes and gene expression, and then investigated if T3 had an attenuating (blocking) effect on these changes (see Table 1 for cut-offs used).

T3 had no discernible effect on the overall differentiation-associated gene expression trajectory of monocytes (Figure 1B). Differentiation-associated DEGs were identified as those that are upregulated or downregulated over time in RPMI medium alone compared to 0 h, with a total of 7604 genes dynamically expressed at 4 h or 24 h in culture. Of these, we identified the 40 differentiation-associated DEGs attenuated by T3 (Figure 1C, Table S1), including toll-like receptor 4 (*TLR4*), which was upregulated by T3 at both time points (Figure 1D, Table S1). These gene lists and their associated gene ontology terms are listed in Table S1.

Unlike gene expression, T3 appears to impede the overall differentiation-associated methylation changes (Figure 1B). We identified 18,510 differentiation-associated differentially methylated probes (DMPs) with a change (gain or loss) in methylation of >5% (*p*-value < 0.05) after 24 h in RPMI culture medium relative to the 0 h control. To investigate the effect of T3 on these DMPs, we focused on the DMPs in which T3 significantly attenuated the methylation change by a magnitude of at least 5% (*p*-value < 0.05). T3 attenuated the methylation change of 312/7210 (4.3%) gain-of-methylation DMPs and 315/11,927 (2.6%) loss-of-methylation DMPs (Figure 1E, Table S2). The differentiation-associated gain-of-methylation promoter DMPs attenuated by T3 were significantly enriched for the biological process terms including “regulation of metabolic process” (*p*-value = 0.0003) (Figure S1A, Table S2), with 63% of probes within this term (Figure S1B). The loss-of-methylation promoter DMPs (DMP < 5 kb from TSS) attenuated by T3 were significantly enriched for “neurotransmitter-gated ion channel clustering” (*p*-value = 0.0006) and a number of catabolism and differentiation processes, as well as the KEGG pathway “steroid hormone biosynthesis” (Figure S2A,B, Table S2). The genomic regions around differentiation-associated promoter DMPs were enriched for the HIF-1α and EGR1 motifs for gain- and loss-of-methylation DMPs, respectively. By contrast, distal DMPs (DMP > 5 kb from TSS) were enriched for the ATF3 and AP-1 motifs, respectively. The strongest differentiation-associated DMPs (and nearest gene) attenuated by T3 are plotted in Figure 1F (ranked by change in methylation (mean Δβ) of T3 vs. RPMI). Additionally, we identified seven differentially methylated regions (DMRs) where T3 attenuates differentiation-associated loss of methylation (at the *LAMTOR2*, *BCL11A*, *ELF1*, *ZNF471*, *TARS2*, *BAZ1B*, and *MPV17L* genes; Figure S2C), and four DMRs where T3 attenuates differentiation-associated gain of methylation (at the *NTNG1*, *PICALM*, *ZEB2*, and *RASGRP3* genes; Figure S1C).

We observed minimal overlap between differentiation-associated DMPs and DEGs attenuated by T3. Only one DEG, *MPEG1*, was associated with a DMP (cg25741594 located 10,016 bp from TSS). Both the gene expression of *MPEG1* and the methylation of cg25741594 were reduced following differentiation, with this effect hindered in the presence of T3.

### 3.2. T3-Specific Remodeling of DNA Methylation in Monocytes

**Figure 2:**
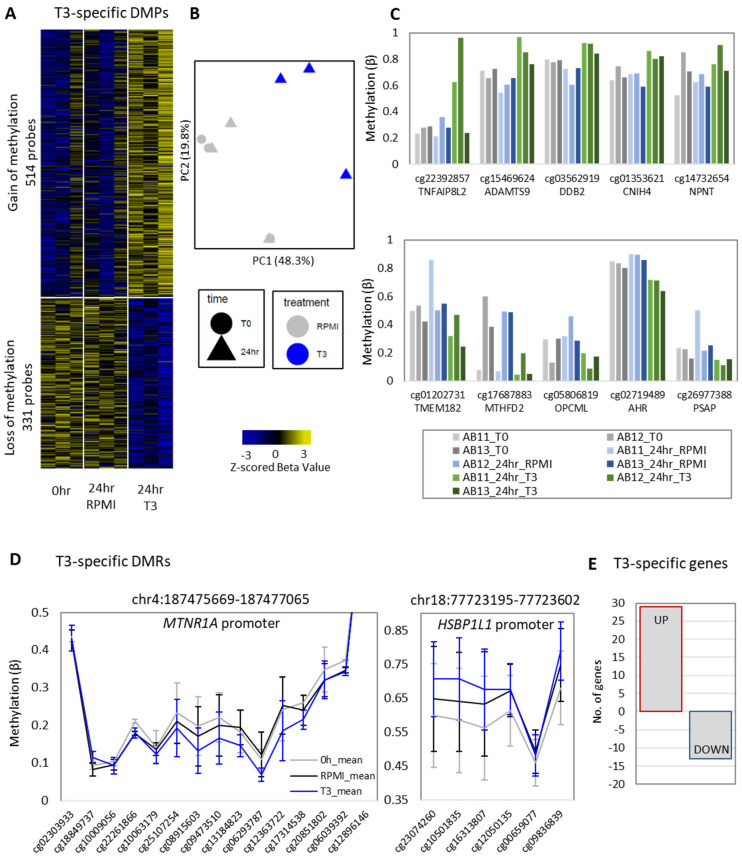
T3 treatment induces a specific methylation signature in human monocytes. (**A**) We identified 845 T3-induced DMPs, with 514 showing a gain of methylation relative to RPMI and 0 h controls (Δβ > 0.05, unadjusted *p*-value < 0.05), and 331 showing a loss of methylation (Z-scored individual donor β values) (**B**) Separation of T3 samples from controls on a PCA plot based on DMPs (separation on PC1). (**C**) Strongest T3-induced gain-of-methylation DMPs (top) and loss-of-methylation DMPs (bottom) and nearest gene. (**D**) An example of a T3-induced loss-of-methylation DMR in the *MTNR1A* promoter and a T3-induced gain-of-methylation DMR in the *HSBP1L1* promoter. Error bars show 95% confidence intervals. For a comprehensive figure of all T3-induced DMRs, see Figure S3C,D. DMRs were identified using DMRcate, with a minimum of 3 CpGs in the DMR, a *p*-value of <0.05, and at least 1 CpG with Δβ > 0.05. DMRs were then intersected to find T3-specific DMRs (i.e., T3-induced relative to both RPMI and 0 h). (**E**) T3 induced a modest number of genes, with 29 upregulated and 13 downregulated genes relative to time-matched RPMI controls and attachment (0 h) (see Figure S3A,B).

To characterize T3-specific effects, we identified altered methylation or expression in T3-treated monocytes relative to both attachment (0 h) and time-matched RPMI-treated monocytes. We identified 845 T3-specific DMPs with a change in methylation of >5% and an unadjusted *p*-value of <0.05 relative to the 24 h RPMI medium control and the 0 h control. Of these, 514 DMPs showed a gain of methylation, and 331 DMPs showed a loss of methylation (Figure 2A, Table S2). T3-specific DMPs could clearly separate T3-treated monocytes from controls in a principal component analysis (PCA) plot on PC1 (Figure 2B). The strongest T3-induced gains and losses of methylation (and nearest genes) are plotted in Figure 2C (ranked by mean Δβ of T3 vs. RPMI). T3-specific promoter DMPs were found in genes enriched for immune-related biological processes, including the “inflammatory response”, “leukocyte activation”, and “complement and coagulation” pathway (Table S2). Additionally, promoter gain-of-methylation DMPs were enriched for hormone processes and pathways, including “estrogen metabolic process”, “steroid hormone biosynthesis”, and “thyroid hormone biosynthesis” (Table S2). Genomic regions around loss-of-methylation promoter DMPs were most enriched for p300 and ETV4 motifs, and gain-of-methylation promoter DMPs were most enriched for the NF-1 half-site motif. By contrast, distal DMPs were enriched for CEBP-β (loss-of-methylation DMPs) and ATF3 (gain-of-methylation DMPs). Several T3-specific DMRs were identified (Figure S3C,D, Table S3) with the strongest DMRs in the promoters of *MTNR1A* (loss of methylation) and *HSBP1L1* (gain of methylation) (Figure 2D).

We identified a small number of T3-specific DEGs: 29 upregulated and 13 downregulated by T3 across both time points (Figure 2E and Figure S3A,B, Table S3). Upregulated genes were most enriched in biological processes “L-serine biosynthesis” and “regulation of lipid metabolism” (Table S3) as well as the GATA and TBP promoter motifs. By contrast, downregulated genes were most enriched in biological processes “regulation of LPS-mediated signaling” and “negative regulation of leukocyte migration” (Table S3) and the SF1 promoter motif.

Again, we observed minimal overlap between T3-specific DMPs and DEGs, with only three DEGs associated with DMPs. These were *ZNF611* (upregulated with T3), which was associated with a gain-of-methylation DMP (cg05660681 located 5,224 bp from TSS); *ARMC9* (upregulated with T3) associated with a loss-of-methylation DMP (cg19262555 located 36,411 bp from TSS); and *ST6GALNAC3* (upregulated with T3) associated with a loss-of-methylation DMP (cg25312440 located 6,831 bp from TSS).

### 3.3. T3 Has a Pronounced Effect on LPS Induced DNA Methylation Remodeling

**Figure 3:**
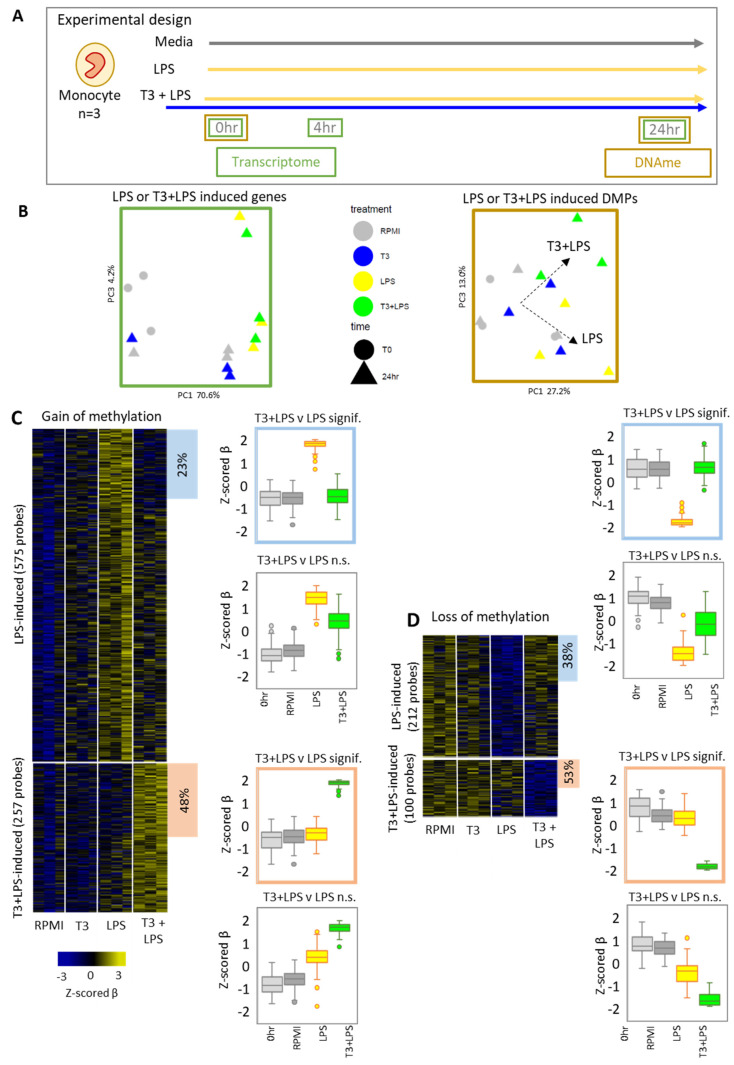
The presence of T3 alters normal LPS-associated methylation and induces a unique costimulation signature. (**A**) Experimental design: Isolated human monocytes (*n* = 3) were exposed in vitro to RPMI medium, 10 ng/mL lipopolysaccharide (LPS), or a costimulation of 5 µM T3 and 10 ng/mL LPS (T3 + LPS). Monocytes were collected at attachment (0 h), at 4 h (for RNAseq), and at 24 h (for RNAseq and DNA methylation). (**B**) PCAs plot of 24 h LPS-upregulated and downregulated genes (left plot, based on 486 unique genes from LPS vs. RPMI and 0 h or T3 + LPS vs. T3 and 0 h comparisons) and of 24 h LPS-induced DMPs (right plot, based on 1133 unique DMPs from LPS vs. RPMI and 0 h or T3 + LPS vs. T3, RPMI and 0 h comparisons). Plots indicate a minimal effect of T3 on LPS-associated expression, but a discernible effect on LPS-associated methylation (on PC3). (**C**) Heatmap of 575 LPS-induced gain-of-methylation probes (LPS vs. RPMI and 0 h, Δβ > 0.05, unadjusted *p*-value < 0.05) and 257 T3 + LPS-induced gain-of-methylation probes (T3 + LPS vs. RPMI, T3, and 0 h) (Z-scored individual donor β values). Results showed that 23.6% (136/575) of LPS-induced gain-of-methylation DMPs had a significant loss of methylation in the T3 + LPS treatment (highlighted in blue boxplot), and 47.8% (123/257) of T3 + LPS-induced gain-of-methylation DMPs had significant loss of methylation in the LPS treatment (highlighted in orange boxplot) (T3 + LPS vs. LPS, Δβ < −0.05 or > 0.05, unadjusted *p*-value < 0.05). (**D**) Heatmap of 212 LPS-induced loss-of-methylation probes (LPS vs. RPMI and 0 h, Δβ > 0.05, unadjusted *p*-value < 0.05) and 100 T3 + LPS-induced loss-of-methylation probes (T3 + LPS vs. RPMI, T3, and 0 h). Moreover, 38.2% (81/212) of LPS-induced loss-of-methylation DMPs had a significant gain of methylation in the T3 + LPS treatment (highlighted in blue boxplot) and 53% (53/100) of T3 + LPS-induced loss-of-methylation DMPs had a significant gain of methylation in the LPS treatment (highlighted in orange boxplot) (T3 + LPS vs. LPS, Δβ < −0.05 or > 0.05, unadjusted *p*-value < 0.05).

Considering that T3 altered the methylation and expression of genes involved in immune processes, including the LPS receptor *TLR4*, we were interested in the effect of T3 on the in vitro monocyte LPS response. To investigate this, isolated monocytes were pre-treated with RPMI medium alone or with 5 µM T3 for 1 h, followed by the addition of 10 ng/mL LPS for 4 h or 24 h (referred to as LPS and T3 + LPS), as shown in Figure 3A. In total, 486 unique genes were upregulated or downregulated in response to 24 h LPS stimulation (LPS vs. RPMI and 0 h, or T3 + LPS vs. T3 and 0 h), and PCA clustering based on these genes showed that the effect of the presence of T3 was minimal (Figure 3B). In contrast, the presence of T3 had a discernible effect on 24 h LPS-induced methylation (gain or loss of methylation in LPS treatment vs. RPMI and 0 h, or T3 + LPS treatment vs. T3, RPMI, and 0 h), with T3 + LPS-treated monocytes showing a different trajectory to LPS-treated monocytes on a PCA plot (PC3) based on these 1133 DMPs (Figure 3B).

Indeed, we observed robust changes to the LPS-induced methylation signature in the T3 + LPS-treated group at 24 h. LPS alone induced a gain of methylation relative to RPMI and 0 h controls (Δβ > 0.05, *p*-value < 0.05) in 575 DMPs (Figure 3C) and a loss of methylation (Δβ < −0.05, *p*-value < 0.05) in 212 DMPs (Figure 3C, Table S4). For gain-of-methylation DMPs, 23.6% (136 probes) had significantly lower methylation in the T3 + LPS treatment at a magnitude of > 5% (T3 + LPS − LPS, Δβ < −0.05, *p*-value < 0.05) (Figure 3C, Table S4). These 136 probes were associated with genes most significantly enriched in the biological processes of “positive regulation of protein tyrosine phosphatase activity” and “negative regulation of Notch signaling pathway” (Table S4). For LPS-induced loss-of-methylation DMPs, 38.2% (81 probes) had significantly higher methylation in the T3 + LPS treatment at a magnitude of >5% (Figure 3D). Genes associated with these 81 probes were most significantly enriched in the biological processes of “multicellular organism development”, “regulation of response to stimulus”, and “cell migration” (Table S4).

### 3.4. T3 and LPS Costimulation Induces a Unique DNA Methylation Signature

Given that the presence of T3 altered the LPS-specific methylation signature, we next explored if T3 and LPS in combination (T3 + LPS) induced a unique methylation signature. First, we identified 257 gain-of-methylation DMPs (Figure 3C) and 100 loss-of-methylation DMPs (Figure 3D) in T3 + LPS-treated monocytes relative to all LPS-absent controls (0 h, T3, and RPMI groups). Of these, 47.8% (123/257) (Figure 3C) and 53% (53/100) (Figure 3D) were also significantly different compared to LPS alone, which we termed a ‘unique’ T3 + LPS signature, as they were differentially methylated relative to all other treatment groups. Unique gain-of-methylation DMPs were associated with genes most enriched for biological processes of “anatomical structure development” and “calcium ion transmembrane transporter activity” (Table S5), and promoter DMPs were most enriched for the GFI1B motif, whereas distal DMPs were most enriched for the Fra1/FOSL1 motif. Unique loss-of-methylation DMPs were associated with genes enriched for “positive regulation of cell proliferation in bone marrow” and “regulation of catalytic activity” among others (Table S5), and promoter DMPs were most enriched for the ETS:RUNX motif, whereas distal DMPs were most enriched for the EWS:ERG and the ERG motifs.

In addition to DMPs, we also identified six DMRs showing T3 + LPS-induced unique gain of methylation (relative to LPS, T3, RPMI, and 0 h controls) associated with *SPG20*, *TENM2*, *RPRD1A*, *IL12B*, and *ADAM29* (Figure S4, Table S5). We also identified four DMRs showing unique loss of methylation associated with *KIAA0319*, *OSBPL9*, *SLC1A3*, and *HMGA2* (Figure S4, Table S5). *IL12B* was also identified as an LPS-induced gene upregulated in the T3 + LPS group at the 4 h time point.

### 3.5. Presence of T3 Has Opposing Effects on the Expression of LPS-Upregulated Genes

**Figure 4:**
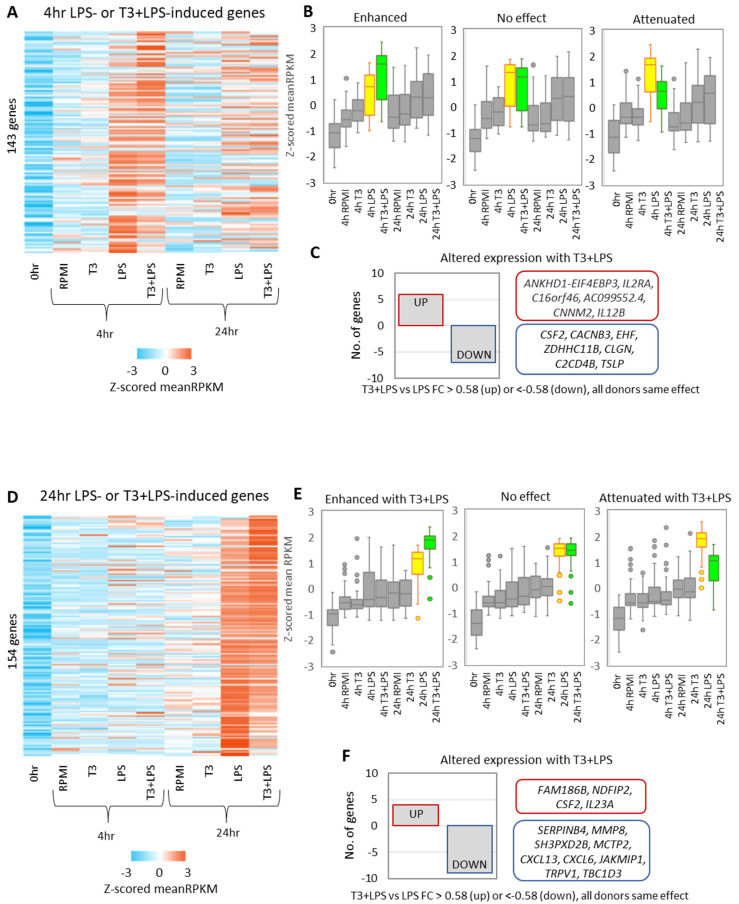
The presence of T3 has opposing effects on expression of LPS-induced genes. Heatmap showing mean expression of 4 h LPS-induced genes (143 genes) (**A**) and 24 h LPS-induced genes (154 genes) (**D**), ranked by the effect of T3 on expression (mean RPKM T3 + LPS/LPS). Gene lists were divided into 3 equal quantiles and Z-scored mean expression values were plotted in boxplots to thus visualizing the opposing effects of the presence of T3 at 4 h (**B**) and 24 h (**E**) (top third = enhanced with T3 + LPS, middle third = no effect with T3 + LPS, bottom third = attenuated with T3 + LPS). Only a modest number of genes were differentially expressed between LPS- and T3 + LPS-treated cells (fold change in mean RPKM > 1.5 with all donors having the same direction of fold change), with 6 and 7 genes upregulated and downregulated at 4 h (**C**), respectively, and 4 and 9 genes upregulated and downregulated at 24 h, respectively (**F**).

In addition to the effect of T3 on the DNA methylation response to LPS, we also investigated the effect of T3 on the transcriptional response. We identified 143 and 154 genes LPS-induced genes (upregulated by LPS relative to RPMI and 0 h, or by T3 + LPS relative to T3 and 0 h) at 4 h (Figure 4A) and 24 h (Figure 4D), respectively (Table S6). We then ranked these genes based on the effect of T3 on LPS response (fold change in mean expression (T3 + LPS/LPS)), and separated the genes into three equal quantiles (top third = T3 enhances LPS response, middle third = T3 has no discernible effect on LPS response, and bottom third = T3 abrogates LPS response) (Figure 4B,E). Ten LPS-induced genes were further upregulated with T3 + LPS and sixteen were downregulated with T3 + LPS (Figure 4C,F, Table S6). Two of these genes were associated with LPS-induced DMPs affected by T3 + LPS. These were *C2CD4B* (induced with LPS but downregulated with T3 + LPS), which is associated with a gain-of-methylation DMP (cg09948769 located 56,569 bp from TSS), and *NDFIP2* (induced with LPS and further upregulated with T3 + LPS), which is associated with a loss-of-methylation DMP (cg20023102 located 238 bp from TSS).

## 4. Discussion

Thyroid hormones have wide-ranging effects on the immune system (reviewed in [11,41]), but their effects on the epigenome and transcriptome of specific human immune cells remain unexplored. In the current study, we demonstrate that in vitro exposure of isolated human monocytes to a high dose of T3 results in modest changes to gene expression and more pronounced effects on DNA methylation in both resting and LPS-stimulated conditions. Approximately 3% of monocyte-to-macrophage differentiation-associated and 27% of LPS-induced DNA methylation was abrogated by the presence of T3. Although the number of T3-induced genes with significantly altered expression was modest, they were enriched for specific biological processes and transcription factor binding motifs, suggesting the specific functions of T3 in monocyte gene regulation.

Monocyte-to-macrophage differentiation and trained immunity is underpinned by metabolic rewiring [3,42]. Considering that thyroid hormones regulate metabolism in many cell types (including skeletal muscle, adipocytes, astrocytes, pancreatic cells, and hepatocytes, as reviewed in [43]), it is not unreasonable to speculate that T3 may elicit effects on metabolism genes in peripheral immune cells such as monocytes. Recently, Chen et al. (2021) demonstrated that T3 induces metabolic rewiring and DNA methylation changes resulting in enhanced pluripotency of human iPSCs, and that this is underpinned by PI3K/Akt signaling [44]. Additionally, T3 has also been shown to signal through the PI3K/Akt pathway in rat myocytes and chick embryo hepatocytes [45,46], influencing nitrous oxide (NO) production and reactive oxygen species (ROS) production, respectively. We found that although T3 elicits changes in a modest number of genes and CpGs in resting monocytes, these were highly enriched for metabolism gene processes, suggesting that T3 may target metabolic pathways. PRDM15 is a transcription factor of the Wnt pathway and is involved in regulating metabolism in human immune cells via the PI3K/AKT/mTOR pathway [47,48]. We found that the PRDM15 motif was enriched both in promoters of differentiation-upregulated genes, where expression was significantly hindered with T3, and in genomic regions around differentiation-associated gain-of-methylation promoter DMPs that showed a loss of methylation with T3. Additionally, we observed a T3-induced loss-of-methylation DMR in the *PIK3IP1* gene, which is a negative regulator of PIK3 [49] (Figure S3D). We identified that DMRs in promoters of late endosomal/lysosomal adaptor MAPK and MTOR activator 2 (*LAMTOR2*) and MPV17 mitochondrial inner membrane protein like (*MPV17L*), where differentiation typically results in a loss of methylation but T3 attenuates this effect. *LAMTOR2* has recently been shown to regulate lipid metabolism and foam cell development in macrophages [50], and *MPV17L* is involved in ROS metabolism. We also observed T3-induced changes in gene expression in specific metabolism pathways. For example, T3 upregulated *PSAT1* and *PHGDH*, which are enzymes in the serine biosynthesis pathway, and *CISH*, *PIBF1*, *ABCB4*, and *GFI1*, which are involved in lipid metabolism (Table S3). In recent years, Rodrigues et al. (2019) demonstrated that serine metabolism is integral for LPS-induced IL1β production in murine peritoneal macrophages [51].

Another key regulator of thyroid hormone signaling is Kruppel-like factor 9 (KLF9). KLF9 has been shown to regulate the downstream effects of T3/TR signaling in human hepatocytes and human pluripotent stem cells [52], and in mouse neuronal cells [53]. It has also been proposed to be implicated in the hematopoietic dysfunction observed in congenital hypothyroidism models [54]. In the present study, we found that T3 upregulated *KLF9* expression in resting monocytes (Figure S3A), suggesting that KLF9 may also be implicated in T3-induced transcriptional regulation in monocytes. However, T3-induced genes were not significantly enriched for the KLF9 promoter motif (data not shown). In summary, we observed T3-induced changes in the expression of several metabolism genes, and differential methylation in or near genes enriched in metabolism processes, but further experiments would be required to determine if these changes result in metabolic alterations at a functional level.

We were also interested in the effect of T3 on the LPS response considering that several mouse studies have proposed a pro-inflammatory role of T3 in macrophages, and that T3 induced altered expression of *TLR4* in resting monocytes (Figure S1). Van der Spek et al. (2018) found that knockdown of the thyroid hormone receptor alpha (TR-α) in a murine macrophage cell line reduced the expression of M1-like markers (*NOS2* and *IL1B*) and enhanced expression of the M2-like marker *ARG1* in polarized macrophages. We did not find any significant changes in *NOS2*, *IL1B*, or *ARG1* following T3 treatment in the present study. Knockdown of type 3 deiodinase, an enzyme that converts T4 to T3, resulted in reduced phagocytosis by bone marrow-derived macrophages and reduced *CSF2* expression in response to in vitro LPS [11]. Interestingly, CSF2 was downregulated at the 4 h time point but upregulated at the 24 h time point. T3 treatment has been shown to have a beneficial effect in LPS-induced endotoxemia in mice [10]. In humans, the relationship between thyroid hormone levels, thyroid receptor expression, and the LPS response appears to be complex and bidirectional, particularly in in vivo studies of endotoxemia. LPS injection (2.5 mg/kg) has been shown to result in a suppressed hypothalamic–pituitary–thyroid axis in mice [55], and severe sepsis and septic shock is sometimes accompanied by thyroid dysfunction in human patients; this is commonly termed “low T3 syndrome” [56]. In the present study, we found that the presence of T3 had both enhancing and attenuating effects on LPS-induced genes, but only a small number were considered differentially expressed. Of the LPS-induced genes identified at the 4 h and 24 h time points, we observed the altered expression of 13 genes (9%) and 17 genes (11%), respectively. Specifically, T3 upregulated the expression of LPS-induced cytokines *IL2RA*, *IL12B*, and *IL23A* and chemokine *CCL15* (but had no effect on well-characterized LPS-induced cytokines *IL1B*, *TNF*, or *IL6*) and downregulated LPS-induced chemokine ligands *CXCL13* and *CXCL6*, and cytokine thymic stromal lymphopoietin (*TLSP*). In resting monocytes, the presence of T3 upregulated *TLR4*, the primary surface receptor for LPS, which may contribute to the altered LPS response; however, further studies are required.

Interestingly, T3 and LPS (T3 + LPS) costimulation induced differential methylation in several hundred probes and several regions (DMRs) relative to all other treatments (including the LPS-only treatment), suggesting that T3 and LPS may act in concert to induce this unique methylation signature. One example of this was the DMR in the promoter region of *IL12B*, where T3 + LPS costimulation results in a gain of methylation relative to LPS-only and control treatments. The expression levels of *IL12B* were also higher in the 4 h T3 + LPS costimulation relative to the LPS-only treatment. Elevated serum IL12 levels have been reported in Graves’ disease patients; however, this was found to be correlated with TSH and T4 levels, rather than T3 [57].The mechanisms underpinning this T3 + LPS costimulation signature remain unclear, but warrant further investigation and replication. We have not validated whether LPS-induced genes exhibiting differential expression or methylation result in differences in protein levels following T3 treatment. To validate this, an ELISA or Luminex panel for cytokines could be performed, and functional assays, such as reactive oxygen species (ROS) production and phagocytosis, could be undertaken. Unlike LPS-induced gene expression, the typical LPS-induced loss of methylation changes were substantially abrogated by the presence of T3 (48% of DMPs significantly abrogated in the presence of T3), with DMPs near genes enriched in the biological processes of stimulus response and cell migration (Table S4).

Although we observed T3-induced changes in gene expression and methylation consistent across all donors, there was poor correlation between DEGs and DMPs. This suggests that the mechanisms underpinning changes in gene expression are likely attributed to mechanisms other than DNA methylation. Other epigenetic mechanisms may include histone modifications, micro-RNA (miRNA), and long non-coding RNA (lncRNA). To further elucidate the processes underpinning T3-induced effects on gene expression, studies investigating the effects of T3 on these mechanisms are warranted.

In this study, we used isolated primary peripheral monocytes, which have been exposed to thyroid hormones in vivo, the concentrations of which were not measured. As a result of this limitation, we treated the monocytes with supraphysiological levels of T3 to maximize our ability to capture T3-induced effects. Considering that we demonstrate that supraphysiological T3 does induce changes in gene expression and methylation, it is worth investigating the effects of a T3 dose that is representative of physiological levels, or of a concentration observed in Graves’ disease patients or in gestational hyperthyroidism. Additionally, all three donors were male and therefore we could not determine whether there were sex-specific effects of T3, as has been shown in other in vitro hormone treatments in human innate immune cells, such as reproductive hormones [58].

## 5. Conclusions

We provide novel evidence that in vitro exposure to T3 can alter the transcriptome and methylome of resting human isolated monocytes, with enrichment of differentiation and metabolism genes. We show that the presence of T3 not only alters the normal LPS-induced DNA methylation signature, but also induces a unique T3 + LPS signature. We highlight that the effects of T3 signaling in immune cells is extended to epigenetic remodeling, which may have implications on immunity in the context of thyroid dysfunction. Due to the poor correlation between DMPs and DEGs, we suggest exploring other epigenetic effects of T3 in future studies.

## Figures and Tables

**Table 1:** Details of RNAseq and DNA methylation (DNAme) cut-offs for identifying DEGs and DMPs.

Comparison	RNAseq Cut-Offs for DEGs	DNAme Cut-Offs for DMPs	Category
RPMI vs. 0 h	Log2 FC (counts) > 0.58 or <−0.58, unadj *p*-value < 0.05, mean RPKM > 1	Δβ > 0.05 or <−0.05, unadj *p*-value < 0.05	Differentiation-associated
Of these T3 vs. RPMI	Log2 FC (counts) > 0.58 or <−0.58, unadj *p*-value < 0.05, mean RPKM > 1 (i.e., 0 h < RPMI > T3, or 0 h > RPMI < T3)	Δβ > 0.05 or <−0.05, unadj *p*-value < 0.05 (i.e., 0 h < RPMI > T3, or 0 h > RPMI < T3)	Differentiation-associated and effect attenuated by T3
T3 vs. RPMI and 0 h	Log2 FC (counts) > 0.58 or <−0.58, unadj *p*-value < 0.05, mean RPKM > 1	Δβ > 0.05 or <−0.05, unadj *p*-value < 0.05	T3-specific
LPS vs. RPMI and 0 h	Log2 FC (counts) > 0.58 or <−0.58, unadj *p*-value < 0.05, mean RPKM > 1	Δβ > 0.05 or <−0.05, unadj *p*-value < 0.05	LPS-induced
Of these T3 + LPS vs. LPS	Log2 FC (mean RPKM) > 0.58 or <−0.58, all donors showing same direction of change	Δβ > 0.05 or <−0.05, unadj *p*-value < 0.05	LPS-induced and LPS-induced effect attenuated by T3 + LPS
T3 + LPS vs. T3, RPMI and 0 h	-	Δβ > 0.05 or <−0.05, *p*-value < 0.05	T3 + LPS-specific
Of theseT3 + LPS vs. LPS		Δβ > 0.05 or <−0.05, *p*-value < 0.05	T3 + LPS-specific (and also distinct from LPS-only samples)

## Data Availability

The data sets generated and analyzed for the current study were deposited in the Gene Expression Omnibus repository with the SuperSeries accession number GSE180694. The SubSeries accession numbers are GSE180693 for RNA sequencing and GSE180692 for DNA methylation data.

## Supplementary Materials

The following supporting information can be downloaded at: https://www.mdpi.com/article/10.3390/biomedicines10030608/s1, Figure S1: T3 attenuates differentiation-associated methylation of CpGs in promoters of metabolism genes. Figure S2: T3 attenuates differentiation-associated methylation of CpGs in promoters of genes involved in cell catabolism and differentiation. Figure S3: T3 induces several DMRs and specific upregulation and downregulation in a small number of genes. Figure S4: A T3 + LPS costimulation induces differentially methylated regions, including in the IL12B promoter. Table S1: T3-attenuated differentiation-associated genes, T3-specific genes, and associated gene ontology; Table S2: T3-attenuated differentiation-associated DMPs, T3-specific DMPs, and associated gene ontology; Table S3: T3-specific DMRs and differentiation DMRs affected with T3; Table S4: LPS-induced DMPs attenuated or unaffected by the presence of T3 (T3 + LPS); Table S5: T3 + LPS-induced DMPs and DMRs; Table S6: LPS-induced genes and the effect of T3 + LPS.
