# Supplementary material for: Triiodothyronine (T3) Induces Limited Transcriptional and DNA Methylation Reprogramming in Human Monocytes
Source: Biomedicines. 2022 Mar 4;10(3):608. doi: 10.3390/biomedicines10030608 (PMC8945024; doi:10.3390/biomedicines10030608)
Supplement: Supplementary file 1 [file biomedicines-10-00608-s001.zip › biomedicines-1585612-supplementary proof/Supplementary_Figures_1-4.pdf]

A Differentiation gain of methylation promoter DMPs attenuated with T3

Positive regulation of cellular process (0.0002)
Regulation of metabolic process (0.0003) (63% of promoter probes)
Regulation of peptidase activity (0.0005)
Negative regulation of cell adhesion mediated by integrin (0.0005)
Signal transduction by p53 class mediator (0.0006)

Th17 cell differentiation (0.0052)
Circadian entrainment (0.0405)

BP

KEGG

B Differentiation gain of methylation DMPs attenuated with T3, '**regulation of metabolic process**'

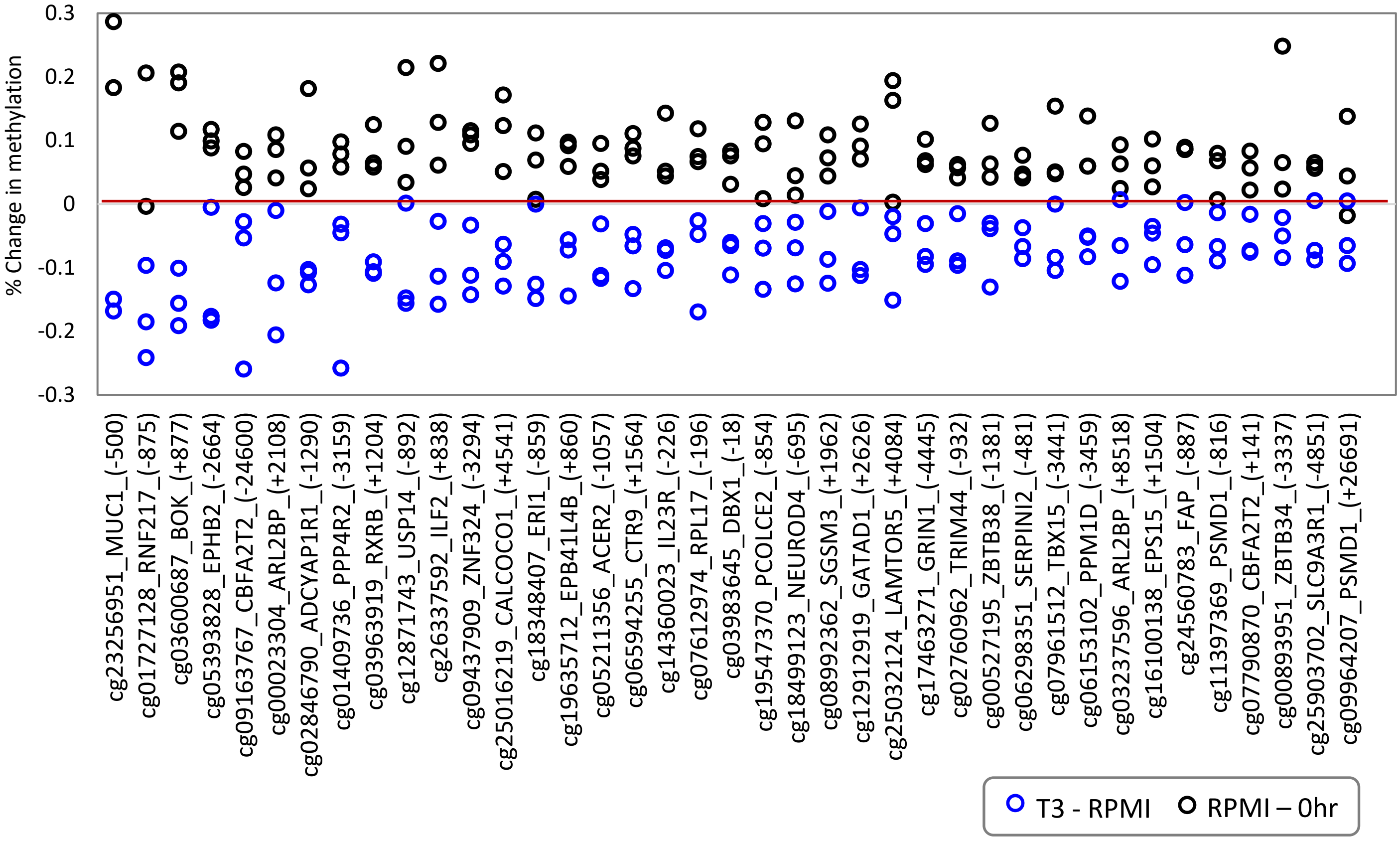

C Differentiation gain of methylation DMRs attenuated by T3

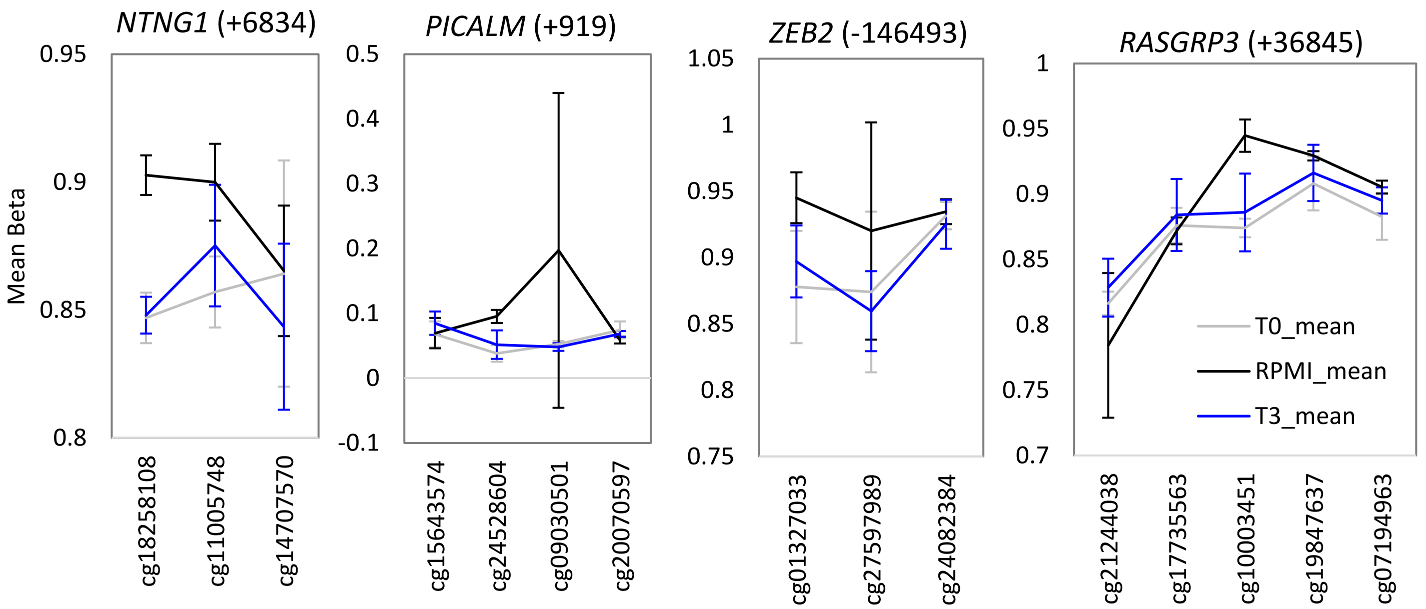

Fig S1

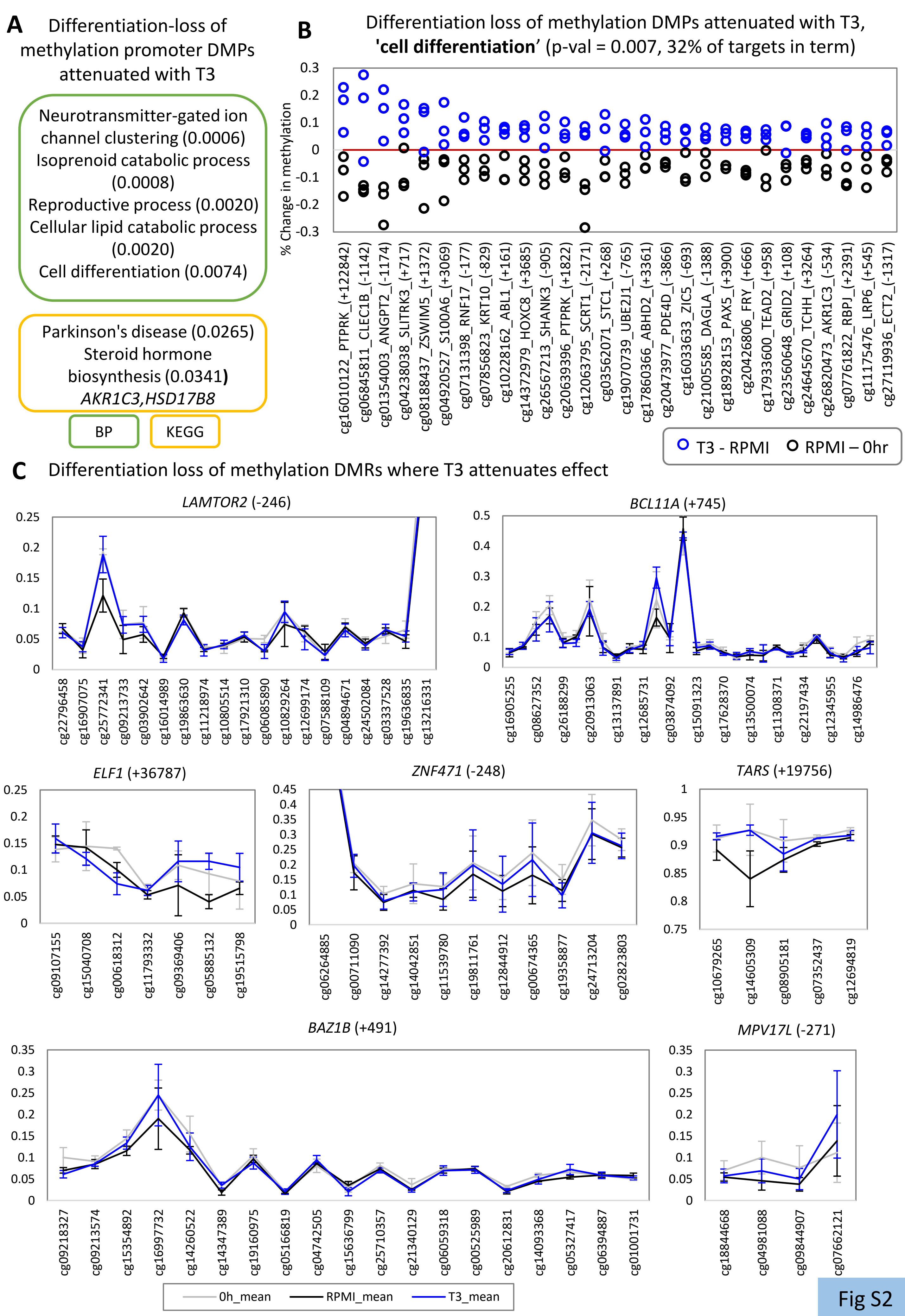
A
Differentiation-loss of methylation promoter DMPs attenuated with T3
Neurotransmitter-gated ion channel clustering (0.0006)
Isoprenoid catabolic process (0.0008)
Reproductive process (0.0020)
Cellular lipid catabolic process (0.0020)
Cell differentiation (0.0074)
Parkinson's disease (0.0265)
Steroid hormone biosynthesis (0.0341)
AKR1C3,HSD17B8
BP
KEGG
B
Differentiation loss of methylation DMPs attenuated with T3, 'cell differentiation' (p-val = 0.007, 32% of targets in term)
% Change in methylation
0.3
0.2
0.1
0
-0.1
-0.2
-0.3
cg16010122_PTPRK_(+122842)
cg06845811_CLEC1B_(-1142)
cg01354003_ANGPT2_(-1174)
cg04238038_SLITRK3_(+717)
cg08188437_ZSWIM5_(+1372)
cg04920527_S100A6_(+3069)
cg07131398_RNF17_(-177)
cg07856823_KRT10_(-829)
cg10228162_ABL1_(+161)
cg14372979_HOXC8_(+3685)
cg26567213_SHANK3_(-905)
cg20639396_PTPRK_(+1822)
cg12063795_SCRT1_(-2171)
cg03562071_STC1_(+268)
cg19070739_UBE2J1_(-765)
cg17860366_ABHD2_(+3361)
cg20473977_PDE4D_(-3866)
cg16033633_ZIC5_(-693)
cg21005585_DAGLA_(-1388)
cg18928153_PAX5_(+3900)
cg20426806_FRY_(+666)
cg17933600_TEAD2_(+958)
cg23560648_GRID2_(+108)
cg24645670_TCHH_(+3264)
cg26820473_AKR1C3_(-534)
cg07761822_RBPJ_(+2391)
cg11175476_LRP6_(+545)
cg27119936_ECT2_(-1317)
T3 - RPMI
RPMI – 0hr
C
Differentiation loss of methylation DMRs where T3 attenuates effect
LAMTOR2 (-246)
BCL11A (+745)
ELF1 (+36787)
ZNF471 (-248)
TARS (+19756)
BAZ1B (+491)
MPV17L (-271)
0h_mean
RPMI_mean
T3_mean

Fig S2

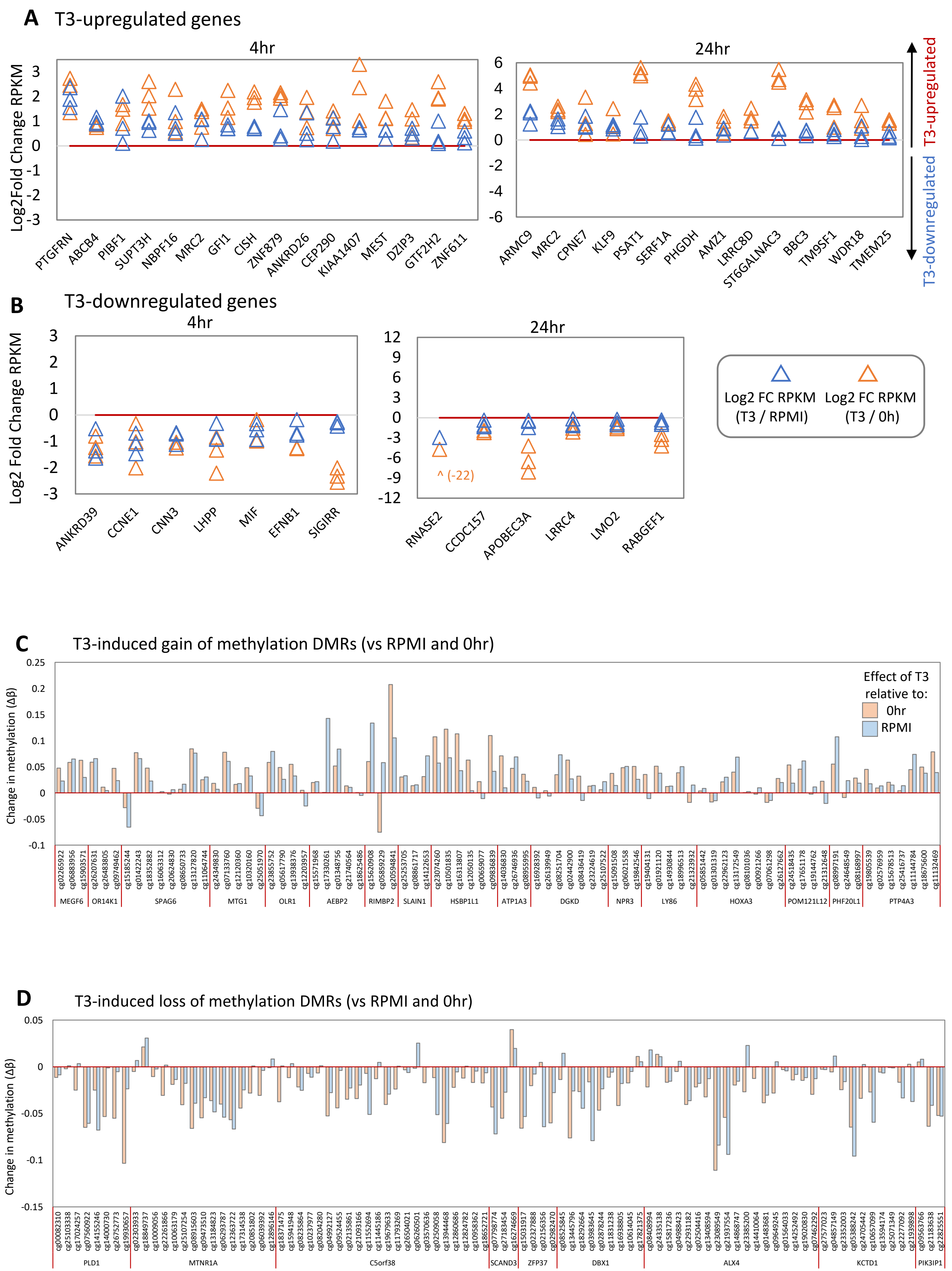
A T3-upregulated genes
4hr
24hr
Log2Fold Change RPKM
PTGFRN
ABCB4
PIBF1
SUPT3H
NBPF16
MRC2
GFI1
CISH
ZNF879
ANKRD26
CEP290
KIAA1407
MEST
DZIP3
GTF2H2
ZNF611
ARMC9
MRC2
CPNE7
KLF9
PSAT1
SERF1A
PHGDH
AMZ1
LRRC8D
ST6GALNAC3
BBC3
TM9SF1
WDR18
TMEM25
T3-upregulated
T3-downregulated
B T3-downregulated genes
4hr
24hr
Log2 Fold Change RPKM
ANKRD39
CCNE1
CNN3
LHPP
MIF
EFNB1
SIGIRR
^ (-22)
RNASE2
CCDC157
APOBEC3A
LRRC4
LMO2
RABGEF1
Log2 FC RPKM (T3 / RPMI)
Log2 FC RPKM (T3 / 0h)
C T3-induced gain of methylation DMRs (vs RPMI and 0hr)
Change in methylation (Δβ)
Effect of T3 relative to:
0hr
RPMI
MEGF6
OR14K1
SPAG6
MTG1
OLR1
AEBP2
RIMBP2
SLAIN1
HSBP1L1
ATP1A3
DGKD
NPR3
LY86
HOXA3
POM121L12
PHF20L1
PTP4A3
D T3-induced loss of methylation DMRs (vs RPMI and 0hr)
Change in methylation (Δβ)
PLD1
MTNR1A
C5orf38
SCAND3
ZFP37
DBX1
ALX4
KCTD1
PIK3IP1
Fig S3

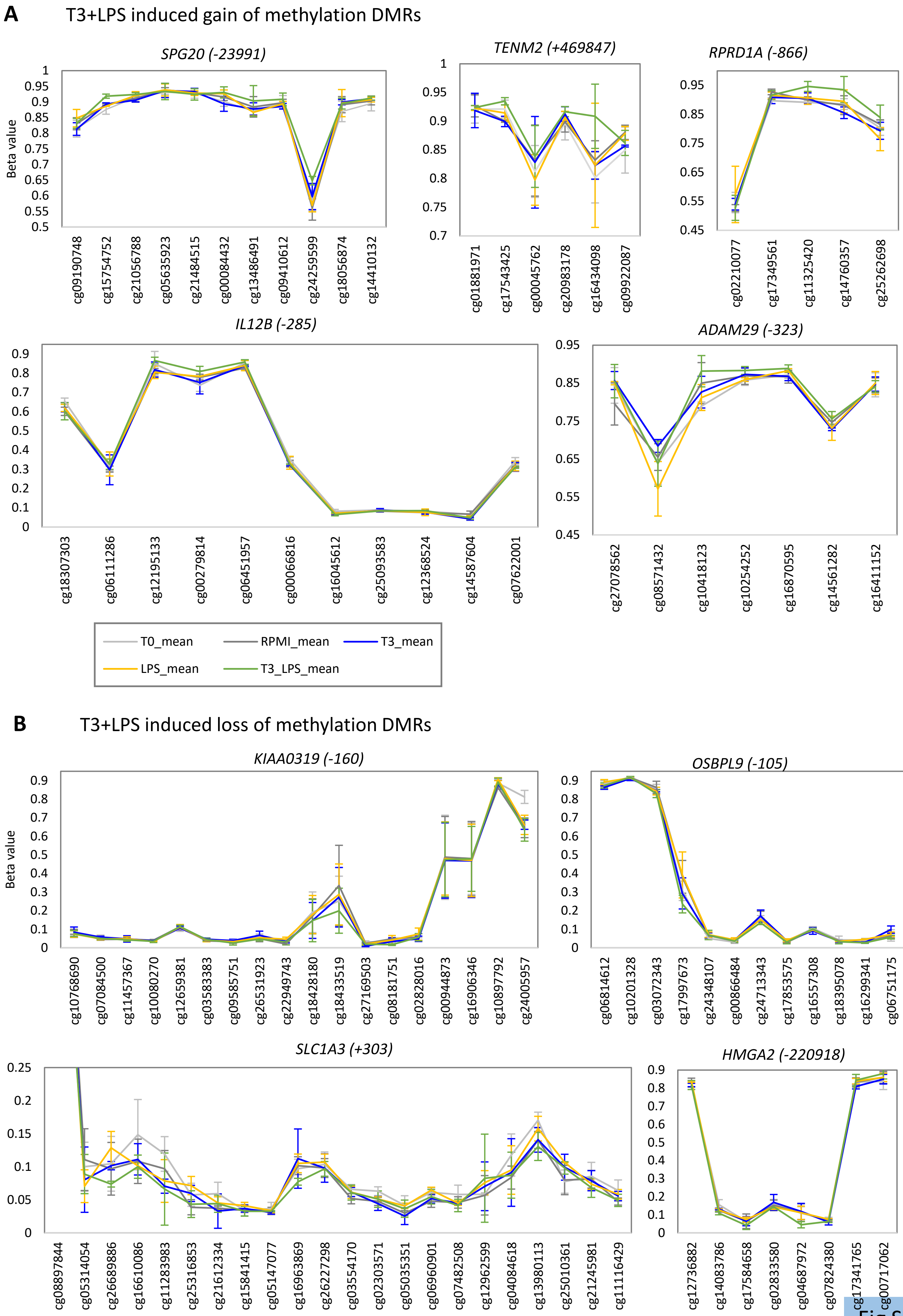
A
T3+LPS induced gain of methylation DMRs
SPG20 (-23991)
Beta value
cg09190748 cg15754752 cg21056788 cg05635923 cg21484515 cg00084432 cg13486491 cg09410612 cg24259599 cg18056874 cg14410132
TENM2 (+469847)
cg01881971 cg17543425 cg00045762 cg20983178 cg16434098 cg09922087
RPRD1A (-866)
cg02210077 cg17349561 cg11325420 cg14760357 cg25262698
IL12B (-285)
cg18307303 cg06111286 cg12195133 cg00279814 cg06451957 cg00066816 cg16045612 cg25093583 cg12368524 cg14587604 cg07622001
ADAM29 (-323)
cg27078562 cg08571432 cg10418123 cg10254252 cg16870595 cg14561282 cg16411152
T0_mean
RPMI_mean
T3_mean
LPS_mean
T3_LPS_mean
B
T3+LPS induced loss of methylation DMRs
KIAA0319 (-160)
Beta value
cg10768690 cg07084500 cg11457367 cg10080270 cg12659381 cg03583383 cg09585751 cg26531923 cg22949743 cg18428180 cg18433519 cg27169503 cg08181751 cg02828016 cg00944873 cg16906346 cg10897792 cg24005957
OSBPL9 (-105)
cg06814612 cg10201328 cg03072343 cg17997673 cg24348107 cg00866484 cg24713343 cg17853575 cg16557308 cg18395078 cg16299341 cg06751175
SLC1A3 (+303)
cg08897844 cg05314054 cg26689886 cg16610086 cg11283983 cg25316853 cg21612334 cg15841415 cg05147077 cg16963869 cg26227298 cg03554170 cg02303571 cg05035351 cg06960901 cg07482508 cg12962599 cg04084618 cg13980113 cg25010361 cg21245981 cg11116429
HMGA2 (-220918)
cg12736882 cg14083786 cg17584658 cg02833580 cg04687972 cg07824380 cg17341765 cg00717062

Fig S4
